# Quality of life after tranexamic acid in subarachnoid hemorrhage: post-hoc analysis of the ULTRA trial

**DOI:** 10.1007/s11136-026-04263-3

**Published:** 2026-05-03

**Authors:** Nadine Denneman, Tanvi Kamra, René Post, Maud A. Tjerkstra, Menno R. Germans, Mervyn D. I. Vergouwen, Korné Jellema, Radboud W. Koot, Nyika D. Kruyt, Jasper F. C. Wolfs, Dharmin Nanda, Bram Van Der Pol, Gerwin Roks, Loes J. A. Reichman, Paul J. A. M. Brouwers, Vincent I. H. Kwa, Henri P. Bienfait, Hieronymus D. Boogaarts, Catharina J. Klijn, René van den Berg, Bert A. Coert, Janneke Horn, Charles B. L. M. Majoie, Gabriël J. E. Rinkel, Yvo B. W. E. M. Roos, W. Peter Vandertop, Dagmar Verbaan, ULTRA trial study group

**Affiliations:** 1 Amsterdam UMC location University of Amsterdam, Neurosurgical Center Amsterdam, Meibergdreef 9, Amsterdam, The Netherlands; 2 Amsterdam Neuroscience, Neurovascular Disorders, Amsterdam, The Netherlands; 3 Department of Neurosurgery, Clinical Neuroscience Center, University Hospital Zurich, Zurich, Switzerland; 4 Neuroscience Center Zurich, University of Zurich, Zurich, Switzerland; 5 Department of Neurology and Neurosurgery, UMC Utrecht Brain Centre, University Medical Centre Utrecht, Utrecht, The Netherlands; 6 Department of Neurology, Haaglanden Medical Centre, Den Haag, The Netherlands; 7 Department of Neurosurgery, Leiden University Medical Centre, Leiden, The Netherlands; 8 Department of Neurology, Leiden University Medical Centre, Leiden, The Netherlands; 9 Department of Neurosurgery, Haaglanden Medical Centre, Den Haag, The Netherlands; 10 Department of Neurosurgery, ISALA Hospital, Zwolle, The Netherlands; 11 Department of Neurosurgery, Elisabeth Tweesteden Ziekenhuis, Tilburg, The Netherlands; 12 Department of Neurology, Elisabeth Tweesteden Ziekenhuis, Tilburg, The Netherlands; 13 Department of Neurology, Ziekenhuisgroep Twente, Almelo, The Netherlands; 14 Department of Neurology, Medisch Spectrum Twente, Enschede, The Netherlands; 15 Department of Neurology, OLVG, Amsterdam, The Netherlands; 16 Department of Neurology, Gelre Hospital, Apeldoorn, The Netherlands; 17 Department of Neurosurgery, Radboud University Medical Centre, Nijmegen, The Netherlands; 18 Department of Neurology, Donders Institute for Brain, Cognition and Behaviour, Radboud University Medical Centre, Nijmegen, The Netherlands; 19 Department of Radiology and Nuclear Medicine, Amsterdam UMC location University of Amsterdam, Meibergdreef 9, Amsterdam, The Netherlands; 20 Department of Intensive Care Medicine, Amsterdam UMC location University of Amsterdam, Meibergdreef 9, Amsterdam, The Netherlands; 21 Department of Neurology, Amsterdam UMC location University of Amsterdam, Meibergdreef 9, Amsterdam, The Netherlands

**Keywords:** EQ-5D, Subarachnoid hemorrhage, Tranexamic acid, Quality of life, Visual analogue scale

## Abstract

**Purpose:**

The ULTRA trial evaluated the impact of ultra-early and short-term tranexamic acid (TXA) treatment in patients with subarachnoid hemorrhage (SAH) and found no clinical benefit at six months. This post-hoc analysis examines whether TXA improves quality of life (QoL) at three and six months.

**Methods:**

The ULTRA trial was a randomized, controlled, multicenter study conducted from July 2013 to July 2019. Patients received either TXA or standard care. This analysis included patients who completed at least one QoL questionnaire. The primary endpoint was QoL, assessed using the EQ-5D-3L questionnaire at three and six months. Linear mixed models adjusted for confounders were used to analyze the association between TXA and QoL.

**Results:**

Of the 955 ULTRA patients, approximately 25% died, and 63% completed at least one QoL questionnaire. At three months, the TXA group had a mean EQ-5D index score of 0.75 versus 0.71 in the control group (*p* = 0.11) and a mean EQ-5D Visual Analogue Scale (VAS) score of 89 versus 86 (*p* = 0.10). At six months, the mean EQ-5D index score was 0.84 in the TXA group compared to 0.82 in the control group (*p* = 0.23), and the mean VAS was 92 in the TXA group compared to 89 in the control group (*p* = 0.13).

**Conclusion:**

Ultra-early and short-term TXA did not result in a significant improvement in QoL at three or six months in patients with SAH. Given the lack of benefit on both clinical outcome and QoL, routine use of TXA is not recommended.

**Trial registration:**

Netherlands Trial Register: NTR3272. ClinicalTrials.gov: NCT02684812.

**Supplementary Information:**

The online version contains supplementary material available at 10.1007/s11136-026-04263-3.

## Introduction

Spontaneous subarachnoid hemorrhage (SAH) is one of the most severe types of stroke and carries a high morbidity and case fatality rate [1, 2]. Survivors frequently experience long-term neurological deficits and cognitive impairment [3, 4]. These have a strong impact on a patient’s ability to function independently [5]. Various studies have also shown that SAH has an adverse effect on self-perceived quality of life (QoL) [6–8].

Recently, the ULTRA trial investigated the effect of tranexamic acid (TXA) treatment on clinical outcome in patients with SAH and found that ultra-early and short-term administration of TXA after SAH does not improve clinical outcome after six months [9].

Although TXA is known to reduce the risk of early rebleeding—a complication strongly associated with poor outcome—this reduction did not translate into improved clinical outcomes in the ULTRA trial [9–11]. This paradox suggests that other pathophysiologic mechanisms may mitigate the expected benefit of TXA. One hypothesized mechanism involves early brain injury, which occurs within the first 72 h following SAH and involves processes such as microthrombosis, inflammation, and neuronal injury [12, 13]. Diffusion-weighted MRI studies demonstrate ischemic lesions in about half of patients during this early phase, even before aneurysm treatment [13, 14]. TXA’s antifibrinolytic effect may decrease the breakdown of microthrombi, potentially worsening microvascular ischemia or delaying recovery from early brain injury [10]. This theory is supported by prior research indicating differential effects of TXA depending on patients’ neurological status, with some evidence of harm in those with impaired consciousness [15]. While TXA is well known for its antifibrinolytic properties and its capacity to promote hemostasis by inhibiting plasminogen activation, the exact pathophysiologic mechanisms by which TXA might influence long-term outcomes after SAH remain incompletely understood, highlighting the importance of evaluating QoL as a complementary outcome, to explore whether TXA might exert subtler effects on recovery and patient well-being beyond clinical endpoints.

QoL assessments offer a comprehensive perspective on a patient’s physical, social, and psychological well-being and overall functioning, which are essential dimensions of recovery which clinical outcomes alone cannot fully capture. Understanding QoL helps to identify the broader impacts of the sequelae of SAH on daily life, including physical impairments, social interactions, and mental health challenges. Despite its importance, QoL assessment is complex due to its wide-ranging nature, which includes various subjective and multifaceted aspects of a patient’s life, requiring robust and nuanced measurement tools [16]. Addressing this gap is vital for ensuring that interventions are truly effective or ineffective in restoring overall well-being and functionality.

The primary aim of this study is to determine whether ultra-early, short-term TXA treatment following SAH influences QoL at three and six months follow-up.

## Methods

### Study design

A complete protocol of the ULTRA-trial has been published elsewhere [17]. In summary, this study was carried out between July 2013 and July 2019, at eight treatment centers and 16 referral hospitals. The trial followed a randomized, controlled, multicenter, open label approach with blinded outcome assessment. The study was performed in accordance with the principles of the Declaration of Helsinki and International Conference of Harmonization guidelines for Good Clinical Practice and was registered on ClinicalTrials.gov (NCT02684812). A description of the informed consent procedure has been published previously [9].

### Patients and procedures

For this post-hoc analysis, we included patients who survived to at least the first follow-up assessment at three months and who met the eligibility criteria of the ULTRA trial [9, 17]. In brief, the ULTRA trial included a total of 955 patients who met the inclusion criteria (age ≥ 18 years, SAH confirmed by CT within 24 h of the most recent hemorrhage). Of these, 480 patients were assigned to the treatment group, receiving standard care per national guidelines along with an intravenous bolus of 1 g TXA, followed by continuous intravenous infusion of 1 g TXA every eight hours for up to 24 h or until aneurysm treatment. The remaining 475 patients were assigned to the control group, receiving standard care alone [18, 19]. Of the 955 enrolled patients, 713 survived the first three months or longer. Written informed consent was obtained from all patients or their legal representatives.

### Outcome

QoL was the primary outcome of this study. QoL was evaluated using the EQ-5D-3L questionnaire [20]. Developed by the EuroQoL group, EQ-5D serves as a standardized measure for quantifying health related QoL [21]. It consists of a self-administered survey including five health dimensions: mobility, self-care, usual activities, anxiety/depression, and pain/discomfort. The EQ-5D-3L classifies health along three levels: no problems (1), some problems (2) and extreme problems (3). A score exceeding 1 suggests the existence of challenges within a specific health dimension. The questionnaire was mailed to the patients’ home address, with instructions to complete it at the three- and six-month follow-up. If no response was received, the research nurse followed up with a reminder phone call. Although patients were permitted to involve proxies in completing the questionnaires, proxies were not actively encouraged to do so on behalf of patients who were unable to complete them. The EQ-5D-3L scoring profiles were converted into index scores using the Dutch EQ-5D tariff [22]. These index scores range from − 0.329 to 1, with 1 indicating optimal health-related QoL. Furthermore, the EQ-5D incorporates a Visual Analogue Scale (VAS), enabling patients to subjectively rate their own health status on a continuum stretching from ‘The best health you can imagine’ (100) to ‘The worst health you can imagine’ (0).

### Statistics

Descriptive data were generated to present the qualitative data. The mean with standard deviation, median with interquartile range (IQR) or number with percentages were used as appropriate to determine the qualitative attributes. Analyses were carried out according to the intention-to-treat principle. A longitudinal dataset was first constructed to facilitate the analysis of repeated measurements. Subsequently, a linear mixed model analysis was performed to evaluate the effect of TXA administration on QoL over time. Fixed effects for time (three and six months) and treatment (TXA vs. control) were included in the model, along with a time-by-treatment interaction term to assess differences in QoL trajectories between both groups. Furthermore, this model allowed for the inclusion of patients with missing data at either the three- or six-month follow-up, as it manages missing data effectively through maximum likelihood estimation. As this study focuses on a subgroup of patients originally randomized in the ULTRA trial, we assessed potential confounding by individually adding several baseline variables to the model. These variables included age, sex, aneurysm treatment (none, endovascular, or clipping), aneurysm location (anterior circulation, posterior circulation, or none), and pre-admission modified Rankin Scale (mRS) – a disability scale ranging from 0 (no symptoms) to 6 (death) [23]. A stratifying variable was deemed a confounder and included in the linear mixed model analysis if the crude and adjusted effects varied by 10% or more. Furthermore, we analyzed whether sex (female/male) and the World Federation of Neurosurgical Societies (WFNS) scale – a clinical severity measure ranging from 1 (Glasgow Coma Scale (GCS) 15 without motor deficit) to 5 (GCS < 7, with or without motor deficit) [24], were possible effect modifiers in the relationship mentioned above. If the interaction term for a potential effect modifier was significant (*p* < 0.05), results of the subgroup analysis were reported. Both unadjusted and adjusted confidence intervals are provided, with the effect estimate (β) representing the adjusted analysis. In addition, we compared the EQ-5D subdomains between the TXA and standard care groups at three and six months and evaluated changes over time. Detailed methods and results of this subanalysis are provided in the Supplemental Material. Finally, a sensitivity analysis including only patients with aneurysmal subarachnoid hemorrhage (aSAH) was performed (see Supplemental Material). Statistical analyses were performed using SPSS version 28 (IBM Corporation, Armonk, NY).

## Results

### Patients

**Fig. 1:**
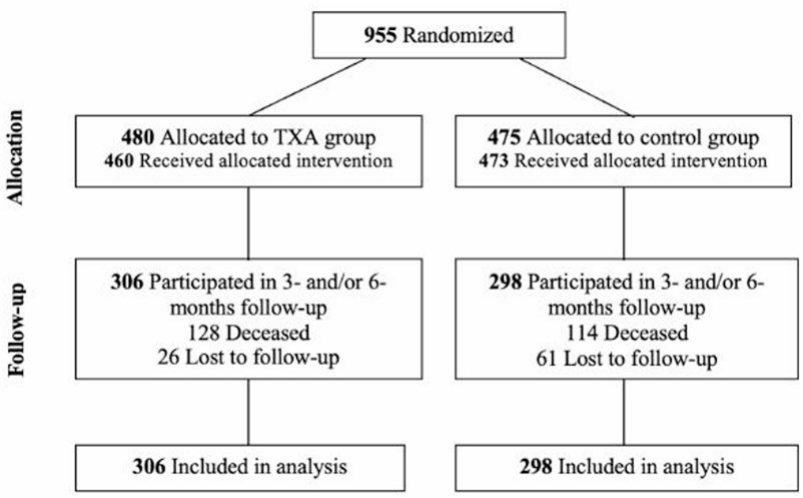
Trial allocation profile of the complete ULTRA cohort. TXA, tranexamic acid

For this post-hoc analysis, 604 of the 713 surviving patients (85%) filled in the EQ-5D questionnaire either at three months, six months, or both and were therefore included. Reasons for exclusion included refusal to participate or unavailability for follow-up. Of the 955 patients initially randomized in the ULTRA trial, 493 (52%) were followed up at three months, and 540 (57%) were followed up at six months (the trial allocation profile of the full ULTRA cohort is shown in Fig. 1). The mean (SD) interval between the ictus and completion of the three-month questionnaire was 101 (18) days, while the interval for the six-month questionnaire was 177 (39) days.

The patients in our study cohort had a mean (SD) age of 58 (11) years and 408/604 patients (68%) were female (Table 1). In 228/306 (76%) patients in the TXA group, and 217/298 (75%) patients in the control group, an mRS of 0 was measured before the initial bleed (indicating absence of any symptoms). In total, in 105 (18%) patients no causative aneurysm was found, and seven patients were classified as ‘Other’ regarding aneurysm location. Within this group, two patients experienced bleeding from a collateral network, one originating from a spinal artery and one originating from a flow-related aneurysm in an arteriovenous malformation. In three patients the exact location of the aneurysm responsible for the bleed could not be determined. In total, 12 (2%) patients did not receive aneurysm treatment. The median length of stay was 18 days.

**Table 1:** Baseline characteristics

Age, in years, mean (SD)	Tranexamic acid group *N* = 306	Control group *N* = 298
	58 (10.9)	58 (11.5)
*Sex*		
Female	216 (70.6)	192 (64.4)
Male	90 (29.4)	106 (35.6)
*WFNS* ^*^		
1	140 (45.9)	146 (48.8)
2	74 (24.3)	74 (24.7)
3	8 (2.6)	6 (2.0)
4	56 (18.4)	44 (14.7)
5	27 (8.9)	28 (9.4)
*Fisher grade score*[25]		
II	28 (9.2)	18 (6.0)
III	102 (33.3)	119 (39.9)
IV	176 (57.5)	161 (54.0)
*Modified Rankin Scale score* ^**^		
0	228 (76.0)	217 (74.6)
1	46 (15.3)	45 (15.5)
2	22 (7.3)	21 (7.2)
3	2 (0.7)	4 (1.4)
4	1 (0.3)	3 (1.0)
5	1 (0.3)	1 (0.3)
*Aneurysm location*		
Anterior circulation	163 (54.0)	161 (54.6)
Posterior circulation	87 (28.8)	81 (27.5)
Other	4 (1.3)	3 (1.0)
None	52 (17.2)	53 (18.0)
*Aneurysm treatment* ^***^		
Endovascular	194 (76.7)	185 (75.5)
Clipping	53 (20.9)	54 (22.0)
No treatment	6 (2.4)	6 (2.4)
Length of stay, median [IQR]	19 [14–28]	18 [14–27]

Characteristics are presented with n and percentages (%) unless stated otherwise. IQR, interquartile range; mRS, modified Rankin Scale score; SD, standard deviation.

^*^WFNS could not be assessed in one patient.

^**^ Modified Rankin Scale score pre-admission was not assessed in 13 patients, and therefore scored as missing.

^***^In 105 patients, no ruptured aneurysm was found. Aneurysm treatment was missing in one patient.

A comparison of baseline characteristics among respondents, non-respondents, and deceased patients is provided in the Supplemental Material (Table S1).

### Outcomes

**Fig. 2:**
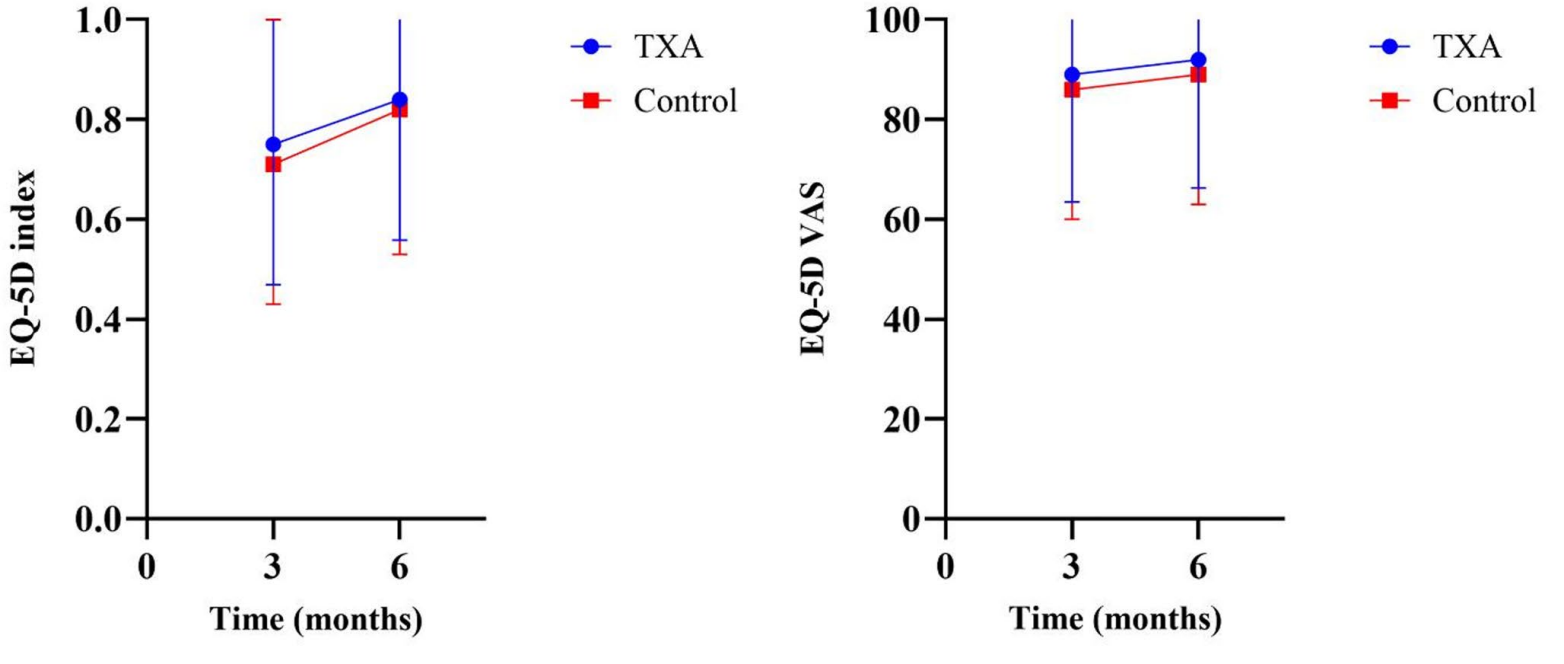
Differences in mean EQ-5D index score and VAS score between TXA and standard care at three and six months. TXA, tranexamic acid; VAS, visual analogue scale.

The primary analysis showed that TXA did not have a significant impact on QoL compared to standard care. Figure 2 illustrates the differences in EQ-5D index scores and self-rated health scores (VAS) for both groups over time. At three months, the TXA group had a mean EQ-5D index score of 0.75 compared to 0.71 in the control group (*p* = 0.11), and a mean VAS score of 89 compared to 86 in the control group (*p* = 0.10). At six months, the mean EQ-5D index score was 0.84 in the TXA group compared to 0.82 in the control group (*p* = 0.23), and the mean VAS was 92 in the TXA group compared to 89 in the control group (*p* = 0.13). A detailed breakdown of the EQ-5D subdomains comparing TXA with standard care, and changes over time, is presented in the Supplemental Material (Table S2).

Table 2 presents the outcomes of the linear mixed model, comparing TXA to standard care in terms of outcome scores at three months, six months, and over time. The adjusted analysis results are presented, along with confidence intervals from the unadjusted analyses. There was no statistically significant time-by-treatment interaction effect, which suggests similar QoL development over time in both groups. The model was adjusted for age, sex, mRS, aneurysm treatment, and location of aneurysm. Furthermore, sex and WFNS were tested for effect modification, but none of the interactions showed a statistically significant difference in effect. The results of the sensitivity analysis focusing on aSAH patients are reported in Supplemental Material (Fig. S1, Table S3, Fig. S2 and Table S4). In summary, TXA did not have a significant impact on QoL in aSAH patients. Furthermore, none of the interactions showed a statistically significant difference in effect within this group.

**Table 2:** Outcome scores of TXA compared to standard care at three and six months and over time

Effect measure	Time	β	Unadjusted (95% CI)	Adjusted^1^ (95% CI)	*P*-value
EQ-5D index score	Intercept	0.71	0.52 · 0.65	0.43 · 1.00	< 0.001
EQ-5D index score	3 months	0.04	− 0.01 · 0.08	− 0.01 · 0.08	0.11
EQ-5D index score	6 months	0.02	− 0.02 · 0.05	− 0.02 · 0.06	0.23
EQ-5D index score	Interaction time * treatment	− 0.01	− 0.05 · 0.02	− 0.05 · 0.03	0.51
VAS	Intercept	86.18	58.85 · 68.70	59.94 · 112.43	< 0.001
VAS	3 months	2.98	− 0.52 · 5.72	− 0.61 · 6.58	0.10
VAS	6 months	2.66	− 1.26 · 4.85	− 0.75 · 6.06	0.13
VAS	Interaction time * treatment	− 0.33	− 3.21 · 1.59	− 3.15 · 2.50	0.82

CI, confidence interval; VAS, visual analogue scale

^1^Model is adjusted for age, sex, modified Rankin Scale score, aneurysm treatment and aneurysm location

## Discussion

In this study, we found that ultra-early and short-term TXA does not enhance QoL at three or six months follow-up in patients with SAH. In both groups QoL increased over time. Additionally, sensitivity analyses showed similar results for aSAH patients.

Previous research highlights the minimal clinically important difference (MCID) as a key measure in QoL questionnaires, alongside statistical significance [26–28]. The MCID is defined as the smallest change in a score that patients perceive as beneficial and that would necessitate a modification in their clinical management. A prior study on stroke patients undergoing rehabilitation estimated the MCID for the EQ-5D index at 0.10 and for the EQ-5D VAS at 8.61 [29]. The between-group changes observed in our trial are considerably lower than the MCID established for stroke patients, indicating that the minimal differences in QoL between the TXA and control groups also lack clinical significance.

Numerous research has consistently highlighted that SAH has an adverse effect on QoL [6–8]. Even a decade after experiencing SAH, health-related QoL of SAH patients remains remarkably lower compared to the general population [30]. However, Greebe et al. found that the well-being of SAH patients tends to improve over time, with notable long-term progress in QoL compared to their QoL shortly after the hemorrhage [31]. These findings align with our results, which indicate an increase in QoL among SAH patients at six months post-hemorrhage compared to three months. Additionally, our study reported EQ-5D index values of 0.84 and 0.82 on average at six months. Considering that the average index score for the healthy Dutch population is 0.87, these values represent a relatively positive outcome for patients recovering from such a severe condition [22]. This may be partly explained by the inclusion of non-aneurysmal SAH patients and the relatively high proportion of low WFNS grades in our population, with 73% of the TXA group and 76% of the standard care group having a WFNS score between 1 and 3 at admission.

There are several strengths to our study. Firstly, this is the first study to investigate the effect of TXA on QoL after SAH. In addition, our study has a very large sample size, and all data were prospectively gathered in multiple centers in the Netherlands over a seven-year period. Lastly, the EQ-5D provides a reliable standardized measure of health-related QoL specifically validated for the Dutch population, making its results more generalizable.

Our study also has a few limitations. The most important limitation of our study concerns missing QoL data and the potential for survivor bias. Approximately one quarter of the original ULTRA cohort died before QoL assessment, and additional patients were unable or unwilling to complete the EQ-5D questionnaires during follow-up. Consequently, patients with more severe neurological deficits or cognitive impairment might be underrepresented in the analyzed sample. The linear mixed-effects models used in this analysis handle missing observations through maximum likelihood estimation, which assumes that data are missing at random (MAR). However, in the context of SAH, missingness may be missing not at random (MNAR), as death or severe cognitive impairment can directly preclude participation in QoL assessments. Violation of the MAR assumption may therefore have introduced bias, possibly resulting in an overestimation of absolute EQ-5D index and VAS scores. Conversely, the opposite effect cannot be excluded. Responder bias may also have occurred, whereby survivors with ongoing needs were more likely to participate than those without such needs, suggesting that patients with excellent recovery and little perceived need for follow-up may have been less inclined to respond [32]. Interpretation of QoL outcomes is further complicated by the disability paradox: patients with favorable functional outcomes do not necessarily report the highest QoL scores, whereas some patients with substantial disability may still report high QoL. This phenomenon has been widely recognized both in the SAH literature and across other clinical fields [33–36]. As a result, the relationship between functional severity, questionnaire participation, and self-reported QoL is complex, and survivor bias does not translate in a strictly linear manner to QoL estimates. Importantly, although these mechanisms may affect absolute QoL levels, they are unlikely to have materially biased the comparative effect estimates between the TXA and control group, given the randomized design and similar follow-up patterns across treatment arms. However, it is possible that treatment bias may have occurred since TXA therapy was not concealed, so group allocation may have been known to patients and treating physicians. Another limitation concerns proxy involvement in the completion of the questionnaires. Although patients were permitted to receive assistance from proxies when completing the EQ-5D, proxy use was not systematically recorded and was not actively encouraged. Consequently, it was not possible to quantify the proportion of proxy-reported questionnaires or to assess the potential influence of proxy reporting on QoL outcomes. Future studies should prospectively document proxy involvement to better evaluate its impact on patient-reported QoL measures. Lastly, it could be argued that the three and six months follow-up period is relatively short, given that some studies on QoL after SAH involved multiple follow-up periods spanning over several years. It is worth mentioning that a six-month timeframe was selected, acknowledging it is a relatively short follow-up period, based on the understanding that the majority of neurological recovery tends to occur within the first six months [37].

The lack of QoL improvement despite TXA’s known effect in reducing early rebleeding parallels findings from the ULTRA trial, which showed no benefit on long-term clinical outcomes despite decreased rebleeding risk [9, 10]. This paradox suggests that other pathophysiologic mechanisms may counterbalance potential benefits.

## Conclusion

This post-hoc analysis of the ULTRA trial shows that ultra-early and short-term tranexamic acid treatment does not improve quality of life in patients with subarachnoid hemorrhage. These findings align with existing evidence suggesting that TXA should not be administered to patients with a subarachnoid hemorrhage.

## Supplementary Information

Below is the link to the electronic supplementary material.

Supplementary Material 1

## Data Availability

The authors have reported all relevant data used to conduct the research. Data are available upon reasonable request by contacting the corresponding author.
