# Supplementary material for: Quality of life after tranexamic acid in subarachnoid hemorrhage: post-hoc analysis of the ULTRA trial
Source: Qual Life Res. 2026 May 3;35(6):148. doi: 10.1007/s11136-026-04263-3 (PMC13136198; doi:10.1007/s11136-026-04263-3)
Supplement: Supplementary file 1 — Supplementary Material 1 [file 11136_2026_4263_MOESM1_ESM.docx]

Supplemental Material

Title: Quality of Life after Tranexamic Acid in Subarachnoid Hemorrhage: Post-Hoc Analysis of the ULTRA trial

Journal: Quality of Life Research

Authors: Nadine Denneman^1,2^, Tanvi Kamra, René Post, Maud A Tjerkstra, Menno R Germans, Mervyn D I Vergouwen, Korne Jellema, Radboud W Koot, Nyika D Kruyt, Jasper F C Wolfs, Dharmin Nanda, Bram Van Der Pol, Gerwin Roks, Loes J A Reichman, Paul J A M Brouwers, Vincent I H Kwa, Henri P Bienfait, Hieronymus D Boogaarts, Catharina J Klijn, René van den Berg, Bert A Coert, Janneke Horn, Charles B L M Majoie, Gabriël J E Rinkel, Yvo B W E M Roos, W Peter Vandertop, Dagmar Verbaan; ULTRA trial study group

Affiliation and e-mail address of the corresponding author:

^1^Amsterdam UMC location University of Amsterdam, Neurosurgical Center Amsterdam, Meibergdreef 9, Amsterdam, The Netherlands

^2^Amsterdam Neuroscience, Neurovascular Disorders, Amsterdam, The Netherlands

n.denneman@amsterdamumc.nl

**Table S1.** Baseline characteristics among respondents, non-respondents, and deceased patients

|  | Respondents  N=604 | Non-respondents  N=114 | Deceased  N=242 | p |
| --- | --- | --- | --- | --- |
| Age, in years, mean (SD) | 57 (11.4) | 55 (13.3) | 63 (13.3) | <.001^1^ |
| **Sex** |  |  |  | 0.70 |
| Female | 409 (67.7) | 73 (64.0) | 164 (67.8) |  |
| Male | 195 (32.3) | 41 (36.0) | 78 (32.2) |  |
| **WFNS^*^** |  |  |  | <.001^2^ |
| 1 | 286 (47.4) | 51 (44.7) | 22 (9.1) |  |
| 2 | 148 (24.5) | 22 (19.3) | 41 (16.9) |  |
| 3 | 14 (2.3) | 7 (6.1) | 10 (4.1) |  |
| 4 | 100 (16.6) | 22 (19.3) | 66 (27.3) |  |
| 5 | 55 (9.1) | 12 (10.5) | 101 (41.7) |  |
| **Fisher grade score[25]** |  |  |  | <.001^2^ |
| II | 46 (7.6) | 8 (7.0) | 2 (0.8) |  |
| III | 221 (36.6) | 23 (20.2) | 36 (14.9) |  |
| IV | 337 (55.8) | 83 (72.8) | 204 (84.3) |  |
| **Modified Rankin Scale score^**^** |  |  |  | 0.22 |
| 0 | 444 (73.5) | 78 (68.4) | 150 (62.0) |  |
| 1 | 92 (15.2) | 21 (18.4) | 18 (7.4) |  |
| 2 | 43 (7.1) | 9 (7.9) | 8 (3.3) |  |
| 3 | 6 (1.0) | 1 (0.9) | 2 (0.8) |  |
| 4 | 4 (0.7) | 1 (0.9) | 4 (1.7) |  |
| 5 | 2 (0.3) | 0 (0.0) | 1 (0.4) |  |
| **Aneurysm location^***^** |  |  |  | <.001^2^ |
| Anterior circulation | 301 (49.8) | 67 (58.8) | 130 (53.7) |  |
| Posterior circulation | 165 (27.3) | 26 (22.8) | 86 (35.5) |  |
| Other | 33 (5.5) | 2 (1.8) | 8 (3.3) |  |
| None | 105 (17.4) | 19 (16.7) | 12 (5.0) |  |
| **Aneurysm treatment^****^** |  |  |  | <.001^2^ |
| Endovascular | 378 (62.6) | 62 (54.4) | 93 (38.4) |  |
| Clipping | 108 (17.9) | 31 (27.2) | 36 (14.9) |  |
| No treatment | 12 (2.0) | 2 (1.8) | 95 (39.3) |  |
| Length of stay, median [IQR] | 18 [14-28] | 23 [14-34] | 6 [1-17] | <.001^3^ |

Non-respondents do not include the deceased patients.

Characteristics are presented with n and percentages (%) unless stated otherwise.

The three groups were compared using a chi-square test for categorical variables and ANOVA for continuous variables.

IQR, interquartile range; mRS, modified Rankin Scale score; SD, standard deviation.

*WFNS score could not be assessed in one respondent and in two patients in the deceased group.

**Pre-admission Modified Rankin Scale (mRS) score was unavailable for 13 patients in the respondents group, four patients in the non-respondents group, and 59 patients in the deceased group.

***Aneurysm location data were missing for six patients in the deceased group.

****Aneurysm treatment data were missing for one patient in the respondents group. Additionally, no ruptured aneurysm was identified in 105 patients overall. In the non-respondents group, 19 patients had no ruptured aneurysm. In the deceased group, 18 patients had no ruptured aneurysm identified, or death occurred before treatment could be initiated.

^1^Tukey post-hoc tests showed that the respondents group differed significantly from the deceased group, and the non-respondents group also differed significantly from the deceased group, while there was no significant difference between the respondents and non-respondents.

^2^Adjusted residuals showed that the largest difference was between the respondents group and the deceased group.

^3^Tukey post-hoc tests showed that all three groups differed significantly from each other.

Note: The total number of patients exceeds 955 (the size of the original cohort) because all deceased patients are included in this table. Some patients died after the three-month follow-up period.

**Distribution of EQ-5D subdomains**

Changes in the five dimensions of the EQ-5D questionnaire (Mobility, Self-Care, Usual Activities, Pain/Discomfort, and Anxiety/Depression) between three and six months were compared between the TXA and standard care groups. To address missing data at follow-up and minimize bias, multiple imputation by chained equations (MICE) was employed. Five complete datasets were generated using Predictive Mean Matching (PMM). PMM was specifically selected to respect the boundaries of the ordinal EQ-5D levels and to robustly handle sparse data in the severe problem categories without mathematical convergence failure. Participant IDs were explicitly excluded from the predictor matrix to prevent overfitting.

For the domains of Mobility, Self-Care, Usual Activities, and Pain/Discomfort, longitudinal analyses were conducted using Cumulative Link Mixed Models (CLMM) for ordinal outcomes. The models included fixed effects for group, time (three vs. six months), and a group-by-time interaction term to evaluate whether the trajectory of scores differed significantly between the groups. A random intercept for participant ID was included to account for the correlated nature of repeated measures within individuals.

Due to extreme data sparsity and a lack of variance in the higher severity levels of the Anxiety/Depression domain (resulting in complete separation), these scores were dichotomized into "no problems" (level 1) versus "any problems" (levels 2–5). This domain was subsequently analyzed using a binary logistic mixed-effects model (GLMM) utilizing the same fixed and random effects structure.

Model estimates and standard errors from the five imputed datasets were pooled using Rubin’s rules. A *p*-value of <0.05 was considered statistically significant. All analyses were performed using R software, utilizing the mice, ordinal, lme4, and broom.mixed packages.

**Table S2.** Distribution of EQ-5D subdomain responses at three and six months by treatment group

| EQ-5D dimension | Model Type | Interaction Estimate (Log-Odds) | Standard Error (SE) | p-value |
| --- | --- | --- | --- | --- |
| Mobility | Ordinal (CLMM) | 0.101 | 0.397 | 0.897 |
| Self-Care | Ordinal (CLMM) | -0.093 | 0.812 | 0.964 |
| Usual Activities | Ordinal (CLMM) | -0.119 | 0.568 | 0.943 |
| Pain/Discomfort | Ordinal (CLMM) | 0.089 | 0.472 | 0.936 |
| Anxiety/Depression | Binary (GLMM) | -0.242 | 0.332 | 0.46 |

Estimates represent the Group × Time interaction term from mixed-effects models run on five multiply imputed datasets (pooled using Rubin's rules). The ordinal models (CLMM) estimate the proportional odds of moving to a worse health state, while the binary model (GLMM) estimates the odds of reporting any problems versus no problems. A positive estimate suggests the standard care group had higher odds of worsening over time compared to the TXA group, though no interactions reached statistical significance (*p* > 0.05).

**Figure S1.** Trial allocation profile for aSAH patients


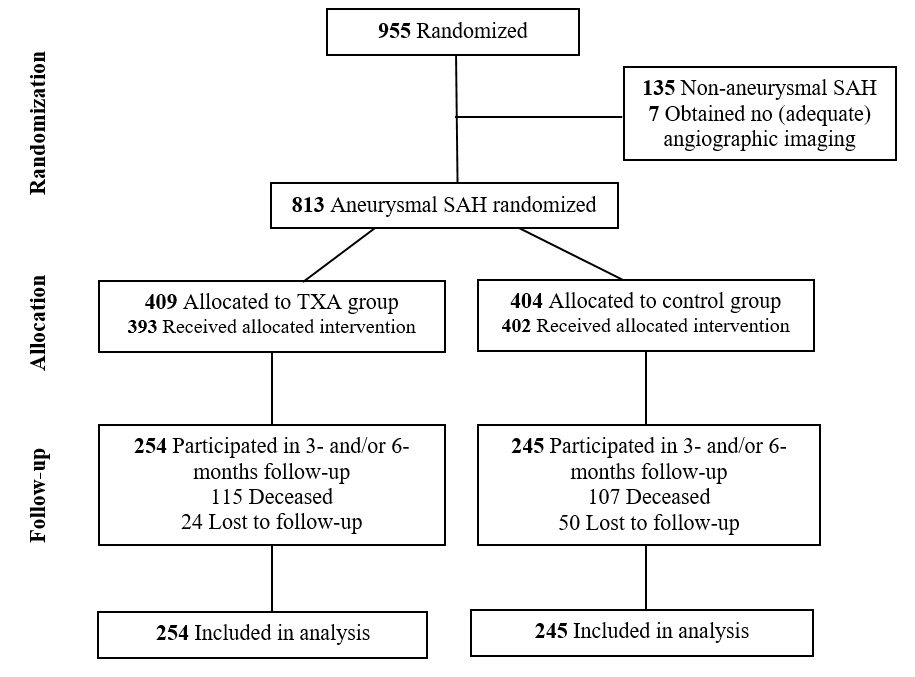
SAH, subarachnoid hemorrhage; TXA, tranexamic acid

**Table S3.** Baseline characteristics for aneurysmal subarachnoid hemorrhage patients

|  | Tranexamic acid group  N=254 | Control group  N=245 |
| --- | --- | --- |
| Age, in years, mean (SD) | 58 (10.6) | 58 (11.7) |
| **Sex, *n* (%)** |  |  |
| Female | 190 (74.8) | 172 (70.2) |
| Male | 64 (25.2) | 73 (29.8) |
| **Fisher grade score, *n* (%)** |  |  |
| II | 20 (7.9) | 11 (4.5) |
| III | 81 (31.9) | 93 (38.0) |
| IV | 153 (60.2) | 141 (57.6) |
| **Modified Rankin Score, *n* (%)^*^** |  |  |
| mRS 0 | 194 (78.2) | 177 (74.1) |
| mRS 1 | 37 (14.9) | 38 (15.9) |
| mRS 2 | 15 (6.0) | 18 (7.5) |
| mRS 3 | 0 (0.0) | 3 (1.3) |
| mRS 4 | 1(0,4) | 2 (0.8) |
| mRS 5 | 1(0.4) | 1 (0.4) |
| **Aneurysm location, *n* (%)** |  |  |
| Anterior circulation | 163 (64.2) | 161 (65.7) |
| Posterior circulation | 87 (34.3) | 81 (33.1) |
| Other | 4 (1.6) | 3 (1.2) |
| **Aneurysm treatment, *n* (%)^**^** |  |  |
| No treatment | 6 (2.4) | 6 (2.4) |
| Endovascular | 194 (76.7) | 185 (75.5) |
| Clipping | 53 (20.9) | 54 (22.0) |
| Length of stay, median [IQR] | 20 [14-29] | 19 [15-29] |

IQR, interquartile range; mRS, modified Rankin Score; SD, standard deviation
^*^ Modified Rankin Score was missing in 12 patients
^**^Aneurysm treatment was missing in 1 patient

**Figure S2.** Differences in mean EQ-5D index score and VAS score between TXA and standard care at three and six months for aSAH


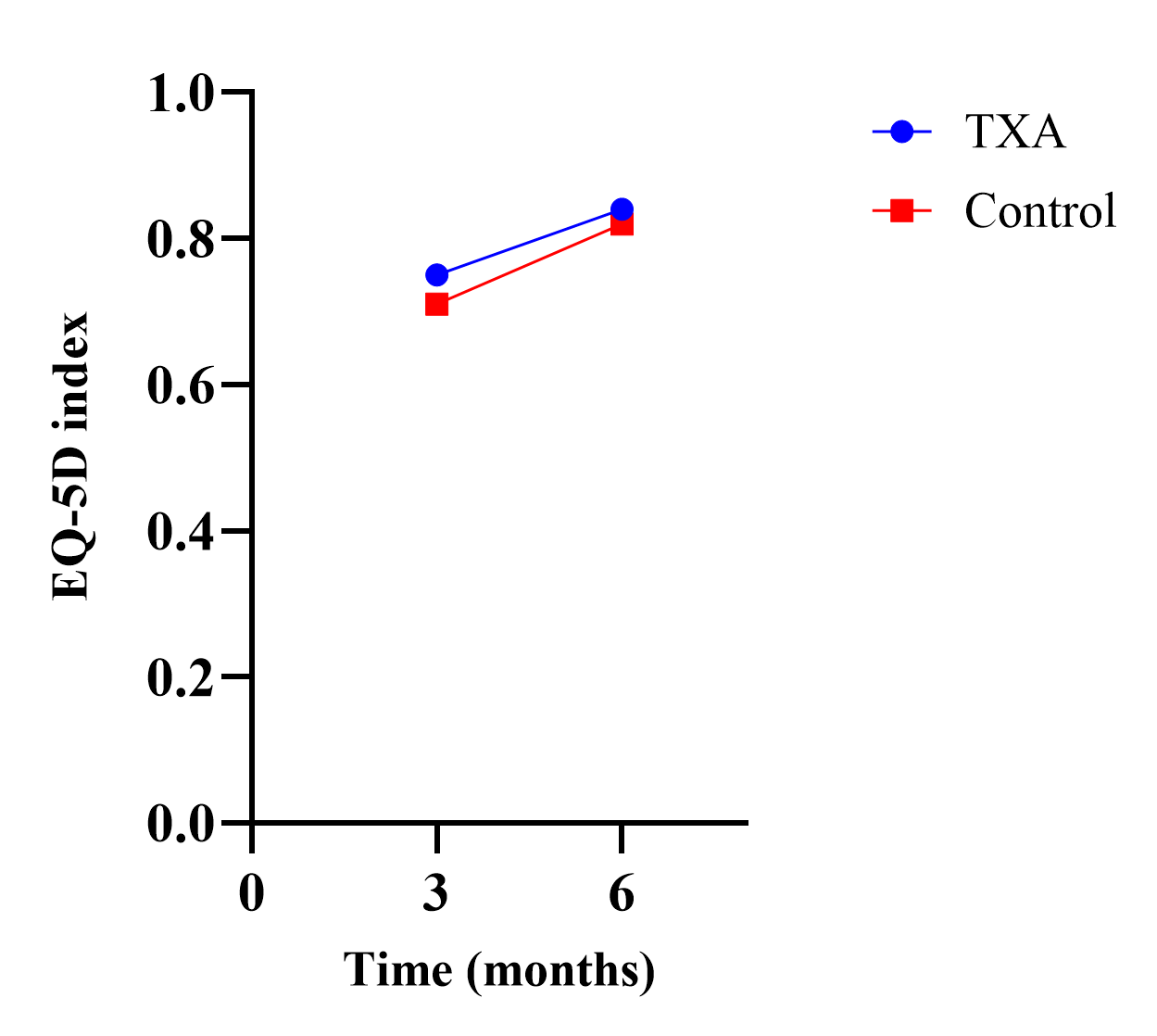

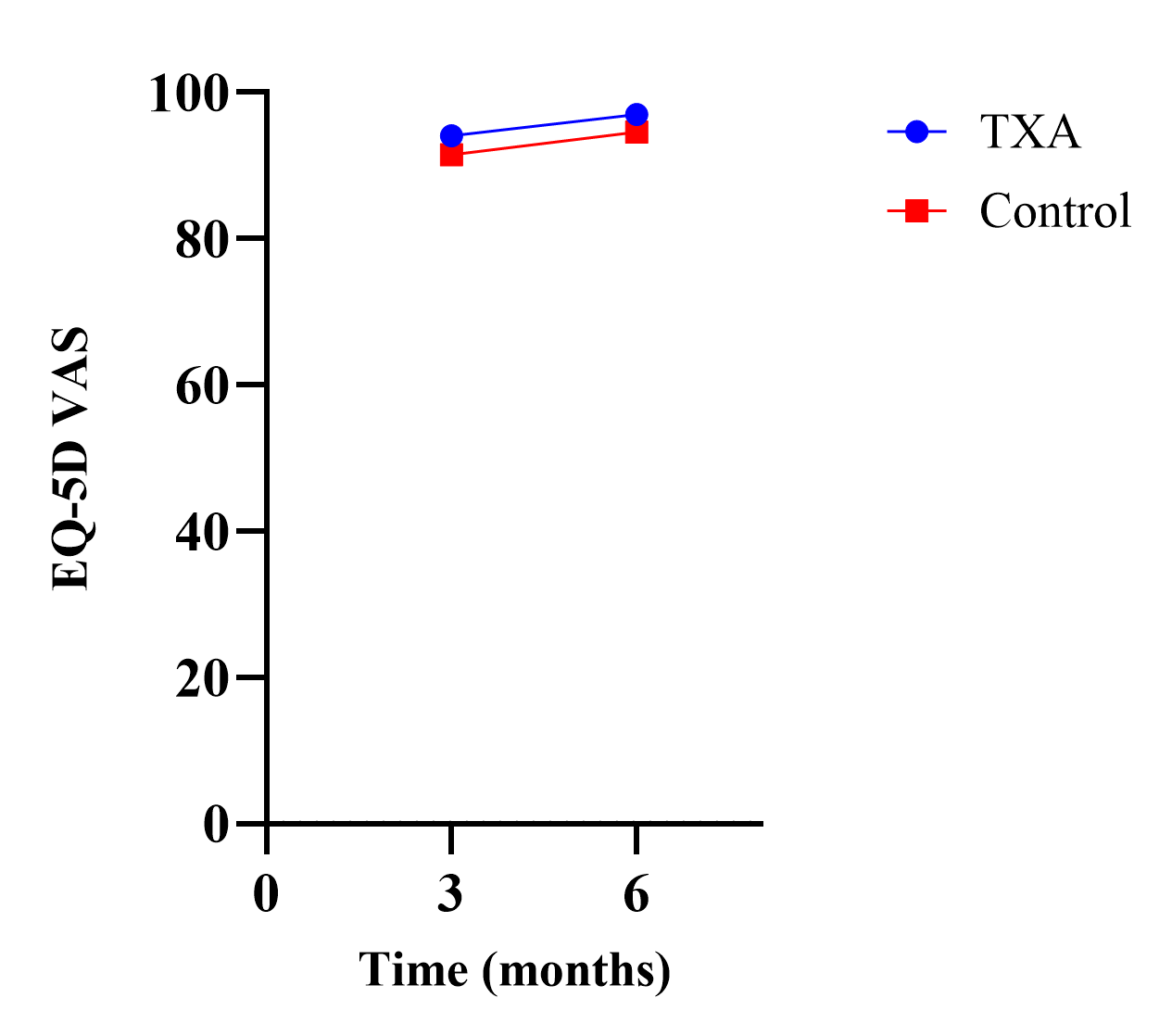


aSAH, aneurysmal subarachnoid hemorrhage; TXA, tranexamic acid; VAS, visual analogue scale

**Table S4.** Outcome scores of TXA compared to standard care at three and six months and over time for aSAH

| Effect measure | Time | β | Unadjusted (95% CI) | Adjusted^1^ (95% CI) | P-value |
| --- | --- | --- | --- | --- | --- |
| EQ-5D index score | Intercept | 0.71 | 0.51 · 0.65 | 0.43 · 1.00 | <0.001 |
| EQ-5D index score | 3 months | 0.04 | -0.01 · 0.08 | -0.01 · 0.08 | 0.11 |
| EQ-5D index score | 6 months | 0.02 | -0.02 · 0.06 | -0.02 · 0.06 | 0.23 |
| EQ-5D index score | Interaction time * treatment | -0.01 | -0.06 · 0.02 | -0.05 · 0.03 | 0.51 |
| VAS | Intercept | 91.46 | 56.25 · 67.49 | 61.03 · 121.88 | <0.001 |
| VAS | 3 months | 2.64 | -0.20 · 6.92 | -0.95 · 6.23 | 0.15 |
| VAS | 6 months | 2.39 | -0.71 · 6. 24 | -1.02 · 5.79 | 0.17 |
| VAS | Interaction time * treatment | -0.26 | -3.38 · 2.18 | -3.08 · 2.57 | 0.86 |

aSAH, aneurysmal subarachnoid hemorrhage; CI, confidence interval; VAS, visual analogue scale

^1^Model is adjusted for age, sex, modified Rankin Scale score, aneurysm treatment and aneurysm location
